# Intersectional discrimination and its impact on Asian American women's mental health: A mixed-methods scoping review

**DOI:** 10.3389/fpubh.2023.993396

**Published:** 2023-02-27

**Authors:** Nicola Forbes, Lauren C. Yang, Sahnah Lim

**Affiliations:** ^1^Applied Developmental Psychology, Department of Psychology, Fordham University, The Bronx, NY, United States; ^2^Department of Biology, New York University, New York, NY, United States; ^3^Department of Population Health, New York University Grossman School of Medicine, New York, NY, United States

**Keywords:** Asian Americans, women, intersectionality, discrimination, gendered racism, mental health, systematic review

## Abstract

**Introduction:**

Gendered racism against Asian American women has become an increasing public health threat in recent years. Although *intersectional* discrimination (i.e., co-occurring race- and gender-based discrimination) against Asian American women is not new, research on this topic is lacking. The present scoping review sought to explore how Asian American women report experiences of intersectional discrimination through a systematic examination of the current literature. We included studies that *explicitly* or *implicitly* discuss intersectional discrimination. We also aimed to identify indicators of psychological wellbeing and coping associated with these experiences.

**Methods:**

Following PRISMA Guidelines for Systematic Scoping Reviews, database searches were conducted for peer-reviewed articles. A total of 1,476 studies were title- and abstract-screened by two independent reviewers. Then, 148 articles were full-text screened for eligibility.

**Results:**

A final sample of 23 studies was identified (15 qualitative and 8 quantitative). Only nine of the included studies explicitly used an intersectional framework. Results from qualitative studies revealed that Asian American women experience intersectional discrimination through fetishization, the ascription of passivity, invalidation through lack of representation and pervasive white beauty ideals, and workplace tokenization and scrutiny. Study findings suggested that Asian American women experience these forms of intersectional discrimination across multiple levels of influence (i.e., internalized, interpersonal, institutional, structural). Findings from both qualitative and quantitative studies also indicated how discrimination, whether explicitly or implicitly intersectional, contributes to adverse mental health outcomes such as body shame, disordered eating, depression, and suicidality. Studies also touched on common coping mechanisms employed by Asian American women when facing or anticipating discrimination, such as avoidance, shifting, proactive coping, and leaning on networks of support. There was a lack of studies using quantitative assessments of intersectional discrimination. Also, most studies did not include disaggregated data by ethnicity, age, sexual identity, religion, socioeconomic status, immigration status, or skin color, all of which are likely to shape their experiences.

**Discussion:**

Our scoping review highlights how the marginalization of Asian American women is an urgent threat to their mental wellbeing. These findings are discussed to inform future research, interventions, and policy changes that prevent racialized and gendered violence against Asian American women.

## Introduction

The deleterious impact of discrimination on the mental health of Asian Americans is well-documented. Higher rates of racial discrimination are associated with increased psychological distress, depression, anxiety, alcohol-related problems, and suicidal ideation among Asian Americans (1–8). Much of the extant research on racism against Asian Americans examines the model minority myth, a common stereotype that characterizes Asian Americans as hardworking, non-political, smart, and invisible (9–11). The model minority myth also perpetuates the dangerous notion that Asian Americans are exempt from experiencing discrimination due to false assumptions of their ubiquitous educational and economic success (9). Another common trope is the perpetual foreigner stereotype. As forever foreigners, Asian Americans are treated like they are not American, often questioned about where they are from, and sometimes viewed as untrustworthy, foreign carriers of disease (9, 12). This “yellow peril” narrative has resurged in the United States (U.S.) since the COVID-19 pandemic (13), and Asian Americans have experienced heightened posttraumatic stress, depression, and anxiety as a result (14–16). Although the model minority myth and perpetual foreigner stereotype shape the experiences of all Asian Americans, research on these forms of racial discrimination often ignores the specific experiences of Asian American women.

Asian American women are burdened with confronting the multiplicative effect of sexism and racism. Sexism has also been found to predict poor mental health, such as depression, anxiety, distress, and disordered eating (17, 18). However, most research on discrimination among Asian American women focuses on race-based discrimination only. Further, most research on sexism has been done with white women, erasing how racism compounds gendered experiences for women of color. For women of color, including Asian American women, experiences of racial discrimination are inextricably tied to gender-based discrimination (19). The interaction of racism and sexism that is experienced by women of color is often known as racialized sexism or gendered racism (20), a form of *intersectional discrimination*.

The intersectionality framework suggests that systems of oppression and power are embedded and reinforced by one another, and the individual experience is shaped through the context of their multiple, interwoven identities (19, 21). In her civil rights legal scholarship, Kimberlé Crenshaw coined the term intersectionality to explicate how Black women's experiences are distinct, and often excluded from, conversations about feminism and anti-racism. Crenshaw has outlined distinctions between structural, political, and representational intersectionality. *Structural intersectionality* is defined as the way macro-level systems shape violence against women of color that is distinct from that of white women (22). *Political intersectionality* is demonstrated through meso-level anti-sexist and anti-racist movements that exclude and further marginalize women of color. For example, feminist movements prioritize the rights of white women and anti-racism often focuses on combatting the oppression of men of color. Thus, these frameworks have traditionally neglected the axis at which women of color exist. *Representational intersectionality* is the way women of color are portrayed, or left out, at the micro-level. Among pioneering Black feminist activists and scholars, such as Sojourner Truth and the Combahee River Collective, intersectionality has driven the conversation on how simultaneously occurring and reinforcing systems of oppression (i.e., racism, classism, heterosexism) have shaped the experiences of Black women in the U.S. for centuries (23, 24). However, the intersectionality framework has only begun to penetrate scholarship within the social sciences (25). Most of the research on intersectional discrimination against Asian American women has been produced within the last decade, and the majority of this research does not utilize an explicit intersectional framework.

Intersectional discrimination against Asian American women is best understood through the history of U.S. imperialism. The positioning of Asian women in the U.S. has been shaped by the intersection of Orientalism and sexism that has pervaded American history for centuries. For example, the Page Law of 1875 legally banned women from China, Japan, or “any Oriental country” from immigrating to the U.S. due to stereotypes of all Asian women as sex workers who would lure white men into a life of sin (12). A few decades later, the War Bride Act of 1945 and the “non-quota immigrants” act of 1946 were implemented to allow Japanese, Korean, and Filipina women who had married American soldiers overseas to migrate to the U.S. (12). This demonstrates the shift from demonizing Asian women to finding them useful as comfort to white men. White sexual imperialism also influenced common cultural tropes to subjugate Asian American women in relation to white men. For example, the “*dragon lady*” (i.e., the hypersexual, deviant Asian temptress) and the “*lotus blossom baby*” or “*China doll”* (i.e., the desperate, hyperfeminine, and sexually servile Asian woman) are particularly prevalent (26–29). Disparaging Asian women to images of subservience and hypersexuality is not only represented in mainstream media but it permeates the pornography world. A content analysis found that Asian women are the most represented female victims within violent pornography and rape websites (30). The racialized sexual objectification of Asian women is reinforced by the alarming rates of sex trafficking of Asian women to the U.S. and contributes to the other types of racialized and gendered violence experienced by Asian American women, such as intimate partner violence and sexual assault (26, 28, 31–33). The murder of six Asian female massage parlor workers in Atlanta in 2021 was a devastating reminder that these intersectional stereotypes exist to empower white men to “eliminate the temptation” of Asian women's bodies (26).

It is critical to acknowledge that discrimination against Asian American women emerges along multiple, simultaneously occurring levels of influence. *Structural* discrimination manifests through macro-level laws, policies, and practices that exclude and subordinate marginalized communities (34). For example, Chinese elders in the New York City metropolitan area experience disproportionately high rates of poverty in comparison to other elders, which is perpetuated and reinforced by barriers at the structural level, like inadequate access to Chinese-language resources and education (35). Cultural ideologies and imagery that dehumanize Asian American women to harmful tropes, like the “*lotus blossom baby*,” are also a product of structural discrimination. At the *institutional* level, Asian American women may experience discrimination through company policies and practices that prevent them from upward mobility in the workplace (36). *Interpersonal* discrimination emerges through interactions between individuals. This is sometimes blatant, such as being called a racial slur, and other times more subtle and regularly occurring acts, often known as “microaggressions” (37–39). Finally, *individual-level*, or *internalized* discrimination, encompasses how Asian American women adopt the stereotypes about them that are perpetuated by these white supremacist, patriarchal systems (9, 40). Importantly, these various levels of influence are reinforced by one another.

The goal of the current scoping review is to assess how Asian American women qualitatively and quantitatively report their experiences of intersectional discrimination, whether explicitly or implicitly, at *all* levels of influence. Our specific aims are to: (1) synthesize studies that look at Asian American women's experiences of intersectional discrimination, regardless of whether intersectional discrimination was the studies' research objective; and (2) examine how experiences of intersectional discrimination are associated with mental health and coping strategies of Asian American women. We hope that findings from the present review can be used to inform future research and tailored interventions and policies to support Asian American women, especially as heightened rates of racialized and gendered violence persist (13).

## Methods

### Inclusion and exclusion criteria

Included articles had to be peer-reviewed, in English, and conducted in the U.S. We included non-experimental, empirical studies using quantitative, qualitative, or mixed methods designs. Data from the studies had to be collected *from* Asian American women. Therefore, studies measuring how *others perceive* Asian American women were not included. Studies assessing *all levels* of influence (e.g., interpersonal, institutional) of discrimination were included. Moreover, studies could be focused exclusively on any subgroup of Asian American women (e.g., South Asian American women, sexual minority Asian American women) or aggregated samples of Asian American women. All studies had to include results on Asian American women's experiences of discrimination. Discrimination was defined inclusively such that a range of more specific terminology could be included (e.g., racism, sexism, racial harassment, ethnic teasing, stereotyping, microaggressions). In addition to including results on discrimination, studies had to fall into one, or both, of the following criteria: (1) mention intersectionality at some point in the article; or (2) include results that speak to intersectional experiences of discrimination. This allowed for both explicitly intersectional (criterion 1) and implicitly intersectional (criterion 2) studies. Finally, studies with samples that were not exclusively Asian American women had to present disaggregated findings on Asian American women to allow for proper data extraction for the current study aims.

### Search strategy

Studies were systematically searched and screened, following the PRISMA Protocol for Scoping Reviews (41). A database search was conducted using EBSCO OneSearch platform of MEDLINE, APA PsycINFO, Gender Studies Database, Gale General OneFile, Gale Academic OneFile, JSTOR, ERIC, JSTOR, SocINDEX, Scopus, APA PsycARTICLES, ScienceDirect, and OpenDissertations for peer-reviewed articles. Reference lists of all included studies were also screened for additional articles. We limited our search to articles published since 2000, based on findings that the first quantitative intersectionality study was published in 2001 (42). The last search was conducted in May 2022. The search strategy is included in Supplementary Table S1.

### Review and data extraction procedure

Duplicate articles were removed prior to screening. Each article was screened by two independent reviewers using Rayyan, a free online software for systematic review study management (43). First, the full sample of articles was title- and abstract-screened by the two independent reviewers. Each independent reviewer excluded articles if they believed they did not meet the inclusion criteria described above. The two independent reviewers then met to reach consensus on articles where there was disagreement on whether they should be included in the full-text screening phase. Then, the two reviewers full-text screened the samples independently, using tags in Rayyan to indicate why an article should be excluded (e.g., tagged as “no intersectionality component”). Again, the two reviewers met to reach consensus on any articles where there was disagreement and come to agreement on the final sample of included articles.

Data was extracted across all included studies and input into Excel tables. Extraction was conducted on general article characteristics (author, title, journal, year), study theoretical framework (intersectionality or other), level of discrimination assessed (structural, institutional, interpersonal, internalized), study aims, and methods (quantitative, qualitative, mixed methods). Given methodological differences in quantitative and qualitative studies, data extraction of study findings was then conducted in separate Excel tables for qualitative and quantitative articles. Among the quantitative studies, a summary of key findings was entered in an Excel table. For qualitative studies, the study team modeled their approach after thematic synthesis, a form of systematic review of qualitative research (44, 45). Emergent themes related to discrimination, mental health, and coping were coded in a data extraction table. Then, an analysis across qualitative studies was conducted on recurring emergent themes that appeared in more than one study. These recurring emergent themes were identified and listed in a separate document. Definitions of commonly emergent themes were created based on findings from the studies. Illustrative quotes from endorsed studies were identified and a count of studies relevant to each theme was calculated. Finally, a synthesis of study characteristics across the full sample of studies was conducted.

### Positionality

The first author is a 29-year-old, biracial, Asian-white cisgender woman (Nicola Forbes). She is in the fourth year of her Ph.D. in developmental psychology. As an Asian presenting woman and having been raised by a Chinese mother and white father, she has always been passionate about understanding the racialized and gendered experiences of Asian women navigating white patriarchal spaces. The second author is a 21-year-old, Asian cisgender woman (Lauren Yang). She is an undergraduate senior at New York University studying biology and on the pre-medicine track. She was born in the U.S. and raised by two Chinese immigrants. She can speak Mandarin, Cantonese, and English. The third author is a 38-year-old, Asian cisgender, bisexual woman (Sahnah Lim). She is an assistant professor with training in public health. She was born in the U.S. and raised in Korea in her formative years and speaks English and Korean fluently. She is experienced in systematic scoping reviews and in conducting research with Asian American women.

## Results

### Overall study characteristics

**Figure 1:**
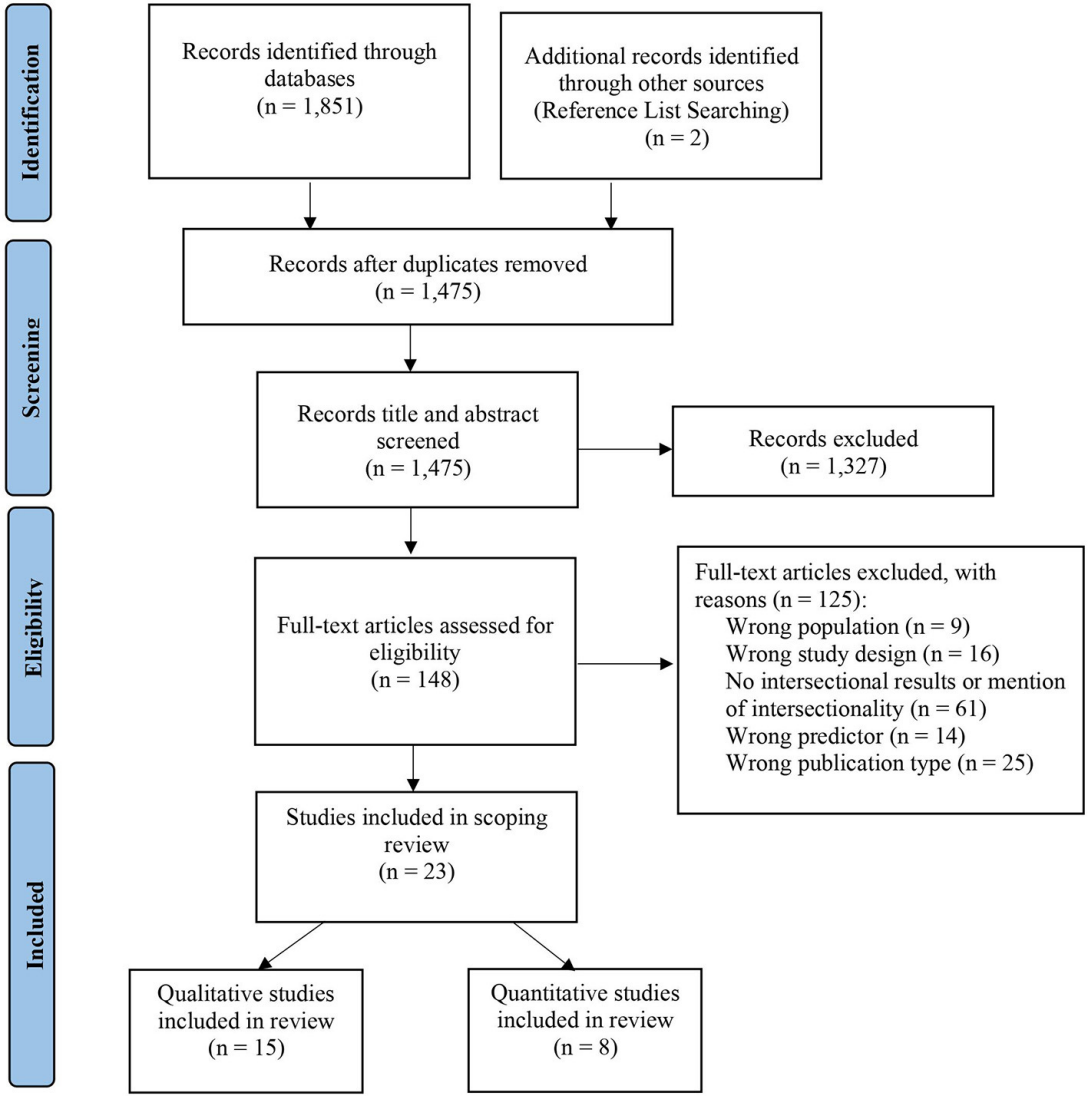
Process of study selection using PRISMA guidelines.

The search yielded a total of 1,475 studies for title and abstract screening. Common reasons for exclusion during the title and abstract phase included wrong publication type, wrong study design, or wrong population. After title and abstract screening, 148 articles met the criteria for full-text review. During full-text screening, “no intersectional results or mention of intersectionality” was the most common reason for exclusion (*n* = 61). The sample resulted in 23 studies examining discrimination experiences among Asian American women, including two that were identified from study reference lists. Of the 23 studies, 15 used a qualitative design and 8 used a quantitative design. There were no mixed-methods studies that met our inclusion criteria. See Figure 1 for the consort diagram.

#### Studies on specific subgroups

Of the 23 articles, 14 were intentionally focused on a specific subgroup of Asian American women (46–59). Four studies examined the experiences of a particular ethnic group, including Japanese American women married to white men (53), Bhutanese-Nepali young women (54), Chinese and Japanese American women (56), and Indian women physicians (46). One study looked at biracial Black-Asian Americans (55). One article was focused on lesbian and bisexual Asian American women (52) and one study was focused on trans and gender diverse Asian Americans (51). Four studies explored the experiences of Asian American women within a specific age group; one included Bhutanese Nepali young women between the ages of 11–24 (54), one examined undergraduate Asian American women between the ages of 18–22 (57), one looked at emerging adult Asian American women (59), and one used a sample of “older” women between the ages of 42–52 (56). Finally, there were five studies exploring Asian American women in the workplace context (46–50). The characteristics of all 23 studies are included in Supplementary Table S2.

#### Intersectional framework

Nineteen of the 23 studies mentioned intersectionality at some point in the study. Nine explicitly used intersectionality as the conceptual framework for their studies. Four of the explicitly intersectional studies were qualitative (47, 50, 57, 58) and five of them were quantitative (60–64). Seven of the studies described intersectionality in the introduction but did not state explicitly that it was the theoretical framework for their study (46, 49, 52, 54–56, 65). Three mentioned intersectionality in the discussion section when drawing on the implications of their findings or suggestions for future research (51, 59, 66). One qualitative study discussed “intersections” without explicitly referring to the intersectionality framework (48). Three qualitative studies never mentioned intersectionality, or “intersections,” at all. However, results on intersectional discrimination emerged across all three, such as themes on the exoticization or fetishization of Asian American women (38, 53, 67).

#### Measure development and validation

Two of the included quantitative studies were scale development and validation studies (61, 63). Both studies utilized an intersectional framework when developing the new scales. One study developed a new measure to assess intersectional microaggressions experienced by Asian American women (63). The Gendered Racial Microaggressions Scale for Asian American Women, a 22-item bifactor model, yielded a general factor and four subfactors: ascription of submissiveness, assumption of universal appearance, Asian fetishism, and media invalidation. The scale was significantly associated with sexism, racial microaggressions, depression, and internalized racism (63).

The second study developed and validated a scale to assess the shifting behavior of Asian American women. Shifting is the process of changing one's appearance, language use, and other behaviors to evade experiences of discrimination (61). The study resulted in the 12-item Asian American Women's Shifting Scale. The scale included three factors: white beauty conformity, bicultural shift, and Asian language/culture avoidance. The scale was significantly associated with subtle racism, blatant racism, gendered racism, and bicultural identity (61).

#### Levels of influence of discrimination

Various levels of influence of discrimination were covered within the breadth of our study sample. Eight studies did not specify the level of influence of discrimination that they aimed to assess but their results suggested multiple levels of influence (46, 49, 50, 52, 54, 57, 58, 65). All eight of these were qualitative studies. Among the other seven qualitative studies, some specified a particular level of influence in their study aims; one aimed to look at *internalized*, or *individual-level*, discrimination (55) and three aimed to identify *microaggressions* specifically (38, 48, 53). Other qualitative studies did not name the specific level of influence until the results section; one qualitative study identified discrimination at the *interpersonal* level in their results (67) and one identified discrimination at the *internalized* and *structural* levels in their results (66). The last qualitative study presented results related to *structural, institutional*, and *interpersonal* discrimination (47). Importantly, even among the qualitative studies that named the level of influence of discrimination in their study aims or results, most studies included findings related to additional levels of influence.

Several of the quantitative studies did not identify the level of influence of discrimination being assessed. However, based on the scales used in the studies, we categorized the levels of influence for each quantitative study. There was less diversity in the range of levels of influence for the quantitative studies. All eight studies included a measure of *interpersonal* discrimination (51, 56, 59–64). Two studies also utilized a measure of *internalized* discrimination (59, 63) and one measured *institutional* discrimination (51).

### Qualitatively defining intersectional discrimination

Findings from the qualitative studies allowed for an exploration of *how* Asian American women are reporting discrimination at the axis of racism and sexism. Several themes emerged across the 15 qualitative studies. The most common emergent themes on intersectional discrimination against Asian American women were collapsed into four categories: (1) *Exoticization, hypersexualization, and fetishization*; (2) *Ascription of the servile and passive Asian woman*; (3) *White female beauty standards and representation*; and (4) *Workplace tokenization and scrutiny*.

#### Exoticization, hypersexualization, and fetishization

The most prevalent theme across studies was the racialized sexualization of Asian American women through the exoticization, hypersexualization, and fetishization of their bodies. Across the 11 studies where this theme emerged (38, 47, 48, 52–55, 57, 58, 65, 67), Asian American women reported feeling fetishized through objectifying and infantilizing comments about their “*doll-like or child-like*” appearances and “*porcelain-like features*” [(57), p. 487]. Asian American women also reported racialized sexual objectification of their body parts by white men, with one participant stating, “*I had a Caucasian boyfriend ask me if my vagina were slanted like my eyes, and then repeat this to his friends. Same boyfriend asked me to use ‘tiny Oriental fingers' to braid his hair for him*” [(65), p. 39]. Participants across studies mentioned being on guard for fetishization: “*I come across a lot of fetishization of Asian, usually in the form of telling me I'm exotic or look like Mulan* [a Chinese female Disney character]. *So, every time I date someone, I have to take into consideration that it might be ‘yellow fever*”' [(52), p. 58]. Japanese women married to white men mentioned being called “*bar girls, prostitutes*,” and told, “*so you know how to please a man*” [(53), p. 185]. Women even reported interpersonal discrimination in the form of racialized sexual harassment while in the workplace (47, 48, 50). For example, one Asian American woman teacher stated that a male colleague told her “*Asian ladies make great wives*” while at work [(48), p. 606].

#### Ascription of the servile and passive Asian woman

Ten studies included findings related to stereotypes of Asian American women as docile, subservient, domestic, and in need of white male saviors (38, 46, 47, 50, 53, 57, 58, 65–67). Some Asian American women reported being socialized by their parents to defy the servile Asian woman trope: “*you should never let the husband fully control you ever*” [(58), p. 8] whereas other families reinforced it through their gendered norms in the home and expectations for women to be self-sacrificing and not complain (66). Some career women were told they were not assertive enough or even lost opportunities for promotion due to being perceived as lacking leadership skills, an ascription of these stereotypes (46, 47, 50, 57, 65).

#### White female beauty standards and representation

An additional but less frequently emergent theme involved commentary on white female beauty ideals and the representation of Asian women in the media. Among three studies, women reported having white beauty standards imposed on them by their family, peers, romantic partners, and the media, such as expectations of being blonde, tall, thin, and light-skinned with large breasts, light-colored eyes, tall noses, and double eyelids (54, 58, 67). Asian American women also discussed the lack of representation or negative representation of Asian women in the media across three studies (57, 58, 67). One participant mentioned that if Asian women were portrayed in the media, it was only as common and problematic stereotypes such as, “*yellow fever in the media or that they are the smart one, quiet, socially awkward, and nerdy*” [(58), p. 9].

#### Workplace tokenization and scrutiny

In addition to the racialized sexist stereotypes that emerged in the workplace, Asian American women also reported tokenization by their superiors and colleagues across five studies (48–50, 53, 65). Women described having excess responsibilities due to being the only Asian woman, the only woman of color, or the only person of color in the workplace. These additional tasks were sometimes centered on diversity efforts or traditionally domestic traits. For example, one teacher mentioned, “*we're always being asked to run the cultural events. Like, the assistant principal approached me and asked if I'd be willing to help lead a cultural potluck*” [(48), p. 613]. Moreover, participants in three studies described being unfairly “under the microscope” (46, 49, 50). Women felt that their work was under heightened scrutiny and that if they made mistakes, they received a harsher scolding than their male and/or white colleagues. Women also described being questioned about their intention to start a family during the job recruitment process: “*I was asked whether I was single, whether I was seeing somebody, whether or not I was serious or married, and if there was any possibility of me having children*” [(46), p. 666].

### Psychological wellbeing and coping: Qualitative and quantitative findings

#### Body image and eating

Several studies assessed how discrimination relates to measures of mental health and coping among Asian American women. Five studies explored how discrimination was associated with body shame or disordered eating (57, 58, 64, 65, 67). One quantitative study found that gendered racial microaggressions predicted disordered eating, but sexism and racism did not (64). This relationship was mediated by body shame, media internalization, and emotion dysregulation. Findings from four of the qualitative studies bolster these quantitative findings (57, 58, 65, 68). Through open-ended responses and semi-structured interviews, Asian American women discussed issues of body surveillance, appearance preoccupation, body dissatisfaction, and even the desire to change their appearance (57, 58, 65, 67). For example, one Indian American woman stated that she felt constant pressure to be thin and while growing up she always heard messages that, “*skinny is the best*” [(67), p. 302]. Importantly, one study mentioned how Asian American women are falsely assumed to not experience body image-related issues because they are expected to be naturally thin (65).

#### Depression and suicidal ideation

Four studies in total examined how discrimination relates to depression and/or suicidal ideation for Asian American women (56, 59, 60, 66). Using latent class analysis among a sample of older women, Chinese American women were most likely to be in the latent class of the highest accumulation of interpersonal discrimination (56). This class was also most likely to report depression. Interestingly, Japanese American women were most likely to be in the latent class of no interpersonal discrimination, which also reported the lowest levels of depression (56). Among a sample of Asian American women, experiences of gender harassment were predictive of more depression. Racial harassment, sexual coercion, and unwanted sexual attention were predictive of posttraumatic stress symptoms (60). However, racial harassment was not significantly associated with depression. Two studies, one quantitative and one qualitative, explored how discrimination experiences relate to suicidality (59, 66). Quantitatively, it was found that gendered racial microaggressions were significantly associated with suicidal ideation and that higher rates of gendered racial microaggressions exacerbated the relationship between self-negativity (i.e., the desire to be white and reject one's Asian identity) and suicidal ideation (59). One qualitative study explored how the model minority myth contributes to the suicidality of Asian American women. Narrative findings demonstrated that Asian American women feel burdened by the pressure to succeed, and some participants reported that past suicide attempts occurred after extreme burnout due to racist, sexist, and intersectional trauma perpetuated in the workplace and the home (66).

#### Coping

Six of the 23 studies sought to identify how Asian American women cope with these experiences of discrimination (49, 52–54, 61, 62). Using the recently developed Asian American Women's Shifting Scale (61), one quantitative study explored the role of shifting in the face of, or anticipation of, discrimination. It was found that shifting mediates the relationship between Asian American identity and Asian American racism-related stress (62). Among the qualitative studies, Asian American women reported a multitude of coping mechanisms. Japanese American women discussed turning to their white husbands for support (53). However, some of them did not find this helpful. One Japanese American woman reported that her white husband perpetuated microaggressions himself. When confiding in her husband about experiencing microaggressions, another Japanese American woman stated that her, “*husband said that she did not have to make ‘a big scene, its nothing'; in this moment*, [participant] *decided, ‘I am divorcing this man*”' [(53), p. 188]. Additionally, Asian American female faculty at a Christian university discussed the following coping strategies: conforming to fit in with their white colleagues, withdrawing and avoiding, and praying (49). Similarly, Bhutanese-Nepali young women discussed finding safe spaces at school where they could be among themselves to avoid being discriminated against by their peers (54). Asian American lesbian and bisexual women discussed conforming to their social contexts, de-emphasizing their sexual minority status, and avoiding situations that could harm them (52). Although many of these coping tactics employ strategies of shifting and conforming to hegemonic culture, several participants across the qualitative studies also highlighted the importance of relying on their social support systems and communities (49, 52–54). Others discussed utilizing empowering strategies such as addressing discrimination or anticipated discrimination in some way (49), calling out discrimination in real-time (53, 54), resisting oppressive norms and engaging in social activism (52).

## Discussion

The present study systematically reviewed the existing research on Asian American women's experiences of discrimination, with a particular focus on *intersectional* discrimination. The review found that Asian American women are commonly hypersexualized and assumed to be passive and docile, experiences that are rooted in white sexual imperialism and cultural stereotypes. Quantitative study findings supported these conclusions. The Gendered Racial Microaggressions Scale for Asian American Women includes several factors that mirror the themes from our qualitative synthesis, such as fetishization and ascription of submissiveness (63). Asian American women also reported being impacted by white beauty ideals and a lack of representation, or misrepresentation, in the media. Importantly, one quantitative study provided support for the correlational relationship between intersectional discrimination, media internalization, and disordered eating habits (64). The included studies also pointed to the role that discrimination plays in other forms of psychological distress, such as depression, posttraumatic stress, and suicidal ideation. However, it is critical to highlight that most of the studies in our sample used qualitative methods. Although qualitative methods provide a direct voice to a population and a clear narrative of their real-life experiences, they are not meant to be widely generalizable. Quantitative designs can assess the size of an effect of a statistical relationship between two variables, such as discrimination and mental health. Quantitative methods are also used to examine whether a correlational relationship is statistically significant or likely due to chance. Thus, there is a particular need to *quantitatively* assess the relationship between *intersectional* discrimination and psychological wellbeing among Asian American women, in addition to gathering rich qualitative data that directly elicits the voices of participants.

Additionally, although examining workplace discrimination was not one of our research aims, several studies emerged that were focused on discrimination in career contexts. More specifically, samples included Indian women physicians (46), Asian American female doctoral students and early career scholars in STEM (47), Asian American women teachers (48), Asian American women in educational leadership (50), and Asian American female faculty at a Christian university (49). Interestingly, workplace discrimination also emerged in several of the non-career studies (53, 65), highlighting how this may be a particularly salient context for the subjugation of Asian American women. It is critical to note that Asian American women were ascribed passive and subservient demeanors by their colleagues and superiors, hindering them from moving up their workplace ladder. This elucidates how Asian American women experience the compounding effect of the *glass ceiling* and *bamboo ceiling* (i.e., metaphorical barriers to preventing women and Asian Americans, respectively, from gaining leadership positions). Together, these findings suggest that even when Asian American women achieve educational and economic success, they are differentially treated in the workplace, dispelling the model minority myth which suggests that Asian Americans do not experience discrimination (9).

Several studies in our sample revealed coping strategies used by Asian American women to prevent and protect themselves from the harmful effects of discrimination. Some participants discussed avoiding situations that could lead to discrimination (49, 54). For others, it was useful to shift their appearance, accent, and cultural orientation to conform to white, hegemonic norms (49, 62). This coping mechanism could be a conscious survival tactic to protect from being excluded and targeted by dominant members of society or demonstrate an internalization of white patriarchal values imposed on them through direct and vicarious discrimination. More research is needed to understand the reasons why Asian American women use shifting to cope. Future quantitative research should make use of the Asian American Women's Shifting Scale (61) to better understand the relationship between shifting and potential risks or protections associated with this coping strategy.

Although both quantitative and qualitative results pointed to the use of shifting, some participants also discussed additional coping mechanisms that highlighted the importance of social support and empowerment. Participants across several qualitative studies reported leaning on their partners or communities for support (49, 52–54). For some, it was important to address discrimination in the moment (53, 54). Importantly, some women also reported empowering themselves by turning to their religion (49) and engaging in social activism (52). There is a particular need for more strengths-based research on effective coping strategies among Asian American women that account for the importance of individual- and community-level empowerment.

Considering the present findings, several major gaps in the literature were identified. First, there is an overall lack of research using an explicitly intersectional framework. Three qualitative studies did not mention intersectionality at all, despite their results pointing to the confluence of racism and sexism (38, 53, 67). Consistent with the literature on the methodological challenges to intersectional research, participants in these three studies discussed intersectional experiences, such as being exoticized and fetishized, despite never being prompted to speak from an intersectional perspective (69). The fetishization and exoticization of Asian women are products of the codified structures that have dehumanized them into sexual objects for white men throughout American history (26, 29). Therefore, researchers within this specialty should take to using an explicitly intersectional framework. When using an explicitly intersectional framework, the reader understands that the scholars were thinking proactively about how multiple interlocking inequities shape the lives of their study population. Further, an explicit intersectional framework requires the scholars to analyze study findings by overlaying historical and political forces, rather than simply presenting results at face value (69).

Furthermore, although there were several studies conducted in the last few years, none of the included studies looked at discrimination related to the COVID-19 pandemic. This is interesting because research from *Stop AAPI Hate* has consistently found that Asian American women are reporting discrimination at significantly higher rates than men since the pandemic began (13). These disparate findings could be due to relatively lower reporting by Asian American men compared to women, or it could be due to the compounding effect of gendered discrimination on anti-Asian discrimination for women. However, research on discrimination during the pandemic has typically not utilized intersectional measures of discrimination. Instead, many have employed quantitative measures of racial/ethnic discrimination (70, 71). This further highlights the need for more intersectional quantitative assessments of the multiplicative effect of racial and gender-based discrimination on Asian American women during the pandemic.

Overall, most of the included studies focused on *interpersonal* discrimination, especially among the quantitative studies. All eight of the quantitative studies measured interpersonal discrimination. This is unsurprising, given that widely used and validated quantitative measures of discrimination often aim to assess interpersonal discrimination, such as the Everyday Discrimination Scale (72). Additionally, the Gendered Racial Microaggressions Scale for Asian American Women is the only existing validated scale measuring intersectional discrimination against Asian American women (63). Several of the included quantitative studies used this scale in their analyses (59, 61, 64). However, this scale is also a measure of interpersonal discrimination, specifically *microaggressions*, further demonstrating the need for more quantitative assessments that capture other levels of influence of discrimination. On the other hand, our qualitative findings pointed to the ways in which Asian American women are disparaged across multiple levels. For example, participants spoke about structural discrimination, such as how the misrepresentation of Asian women in the media impacts their self-esteem (54, 58, 67). Qualitative findings should be used to influence the development of more quantitative measures of intersectional discrimination, particularly at the structural, institutional, and internalized levels. It was also interesting that many of the qualitative studies aimed to assess one level of influence of discrimination, but results pointed to various, or all, levels of discrimination based on respondents' narratives. This highlights the utility of qualitative research in unearthing how Asian American women are experiencing *simultaneously* occurring, multiple levels of intersectional discrimination.

There are several limitations within our scoping review. Our review was limited to the intersections of racism and sexism to understand Asian American women's experiences. This does not represent the additional axes of marginalization that Asian American women experience, such as classism, colorism, homophobia, and transphobia. Moreover, due to the largely heterosexual samples of our studies, the present review was written from a heteronormative lens that largely represents white men as the perpetrators of discrimination. However, white women also benefit from dehumanizing women of color (22). For example, sexual minority Asian American women report being fetishized by white women too (52). Sexual and gender minoritized Asian American women and femmes may also be subjected to intersectional discrimination that is perpetrated by cisgender-heterosexual Asian American women. Thus, there is especially a need for more research on the experiences of intersectional discrimination among sexual and gender minority Asian American women and femmes who are impacted by reinforcing heterosexist, racist, and transphobic systems.

We were also limited in our ability to present findings on specific subgroups due to the lack of disaggregated data in our samples. “Asian American” was used broadly to include American women of any Asian descent. Disaggregation of the sample data was not within the scope of the review, and some included studies did not provide enough demographic characteristics of the study sample. Most samples included largely East Asian, heterosexual, and college-educated Asian American women, misrepresenting the diversity of Asian American women's experiences. For example, one study using latent class analysis found that Japanese American women and Chinese American women belonged to different latent classes based on the frequency of their experiences of discrimination (56). This highlights the unique experiences of different subethnic groups of Asian American women.

Additionally, we did not assess variation in results by age, class, education, skin tone, religion, immigration status, or sexual orientation. In our sample, one study examined discrimination among trans and gender diverse Asian Americans (51). Findings showed that transwomen reported elevated rates of unequal treatment in comparison to other trans and gender diverse Asian Americans (51). Further, several studies utilized samples within specific developmental stages, particularly adolescence and young adulthood (54, 57). Asian American women in their adolescence and young adulthood use higher rates of social media, where interpersonal discrimination is common (58, 73, 74). They are also at the peak of identity development and may be more impacted by the negative psychological consequences of discrimination (75). These studies suggest that Asian American women of various ages may have distinct experiences of intersectional discrimination and the mental health consequences associated with it. However, due to a lack of studies with disaggregated data and specific populations, we were unable to draw conclusions on the experiences of subgroups of Asian American women. More research is necessary to better understand the diversity in experiences of Asian American women across various intersections.

Findings from the present study have several important implications. First, clinicians working with Asian American women should use an intersectional framework to assess how their experiences of oppression and marginalization have shaped their mental health and coping mechanisms. For example, for patients suffering from body shame or disordered eating, it is important to work with them to decolonize and deconstruct their internalization of white female beauty ideals. Similarly, our study findings can be applied to modify workplace diversity practices that prioritize the hiring of men of color and white women over that of women of color. Implementing structural changes to workplace hiring committees that would advocate for hiring and promoting Asian American women, and other women of color, can help to eliminate existing inequities in the workplace. Given reports of intersectional interpersonal discrimination in the workplace, all organizations should require diversity, equity, and inclusion training to teach white colleagues about cultural humility in the workplace. Proper grievance procedures to report gendered racism should also be implemented in all working contexts to ensure that there are accountability structures for perpetrators of intersectional discrimination.

The results from the present study should also be applied to inform healing spaces, interventions, and policies to support Asian American women and their psychological wellbeing. Findings from the studies that examined coping mechanisms demonstrated that many Asian American women use strategies of shifting to conform to white patriarchal norms. However, Asian American women should not have to modify who they are to protect themselves. Instead, we need more feminist decolonization spaces where Asian American women can process, disentangle, and recover from internalized oppression within a safe environment. These spaces should exist within schools, workplaces, and other institutions, and be made accessible for women of all socioeconomic backgrounds. It is critical to note that existing white supremacist, patriarchal systems within the U.S. are responsible for the continued violence against Asian American women at all levels of influence. Thus, it is not the responsibility of Asian American women to improve and challenge these oppressive structures alone. However, Asian American women should be encouraged to find empowerment to cope with the current systems. Interventions to support Asian American women should center on building community, connection, and healing practices. As a society, we must continue to deconstruct the stereotypes that exist to harm this population. Finally, we must eliminate systemic and structural barriers that disproportionately impact all women of color and create policies that champion and protect them from gendered and racialized ridicule and violence.

## Conclusion

This study examined how Asian American women experience and report intersectional discrimination and psychological wellbeing. Through a synthesis of the existing literature, we found that Asian American women are hypersexualized, ascribed as submissive, report having to meet white beauty ideals, and are misrepresented in the media. Several studies also highlighted how Asian American women face direct and indirect discrimination in the workplace. Additionally, included studies examined how intersectional discrimination against Asian American women contributes to their poor mental health outcomes such as depression, suicidal ideation, negative body image, and disordered eating. Asian American women reported a range of strategies to cope with these experiences, such as shifting to dominant norms, avoiding harmful contexts, finding social support, and engaging in activism. Although our review was able to identify 23 studies that met inclusion criteria, there is still a need for future research using an explicitly intersectional framework when examining the experiences of Asian American women. Nonetheless, the present findings point to the concurring, harmful forces of racism and sexism that shape the psychological health of Asian American women across different contexts and various stages of life. Importantly, Asian American women have reported disproportionately high rates of hate incidents and discrimination since the start of the COVID-19 pandemic, suggesting they may be at increased risk for psychological distress. However, they remain an understudied and underserved population. Therefore, findings from the present review highlight the urgent need for future research on this population and the need for increased funding for tailored interventions that support and uplift this population through a feminist and decolonial framework. Findings from this study should also be used to inform institutional, policy, and system-level changes to protect, heal, and empower Asian American women and other women of color.

## Data availability statement

The original contributions presented in the study are included in the article/Supplementary material, further inquiries can be directed to the corresponding author.

## Author contributions

NF was responsible for study conceptualization, article screening and review, data extraction, and writing. LY was responsible for article screening and review and manuscript review. SL was responsible for study conceptualization and writing. All authors contributed to the article and approved the submitted version.
