# Supplementary material for: Intersectional discrimination and its impact on Asian American women's mental health: A mixed-methods scoping review
Source: Front Public Health. 2023 Feb 27;11:993396. doi: 10.3389/fpubh.2023.993396 (PMC10008964; doi:10.3389/fpubh.2023.993396)
Supplement: Supplementary file 1 [file Table_1.docx]

**Supplemental Table 1 Boolean Search Strategy**

| Asian American, Asian, East Asian, South Asian, Southeast Asian, AAPI, OR Asian American Pacific Islander | AND |
| --- | --- |
| racial* sexism, gender* racism, racial* violence, gender* violence, sexual* racism, intersectional discrimination, intersectional stigma, intersectional prejudice, racism, sexism, misogyny, prejudice, microaggress*, discrimination, stigma, stereotyp*, oppress, objectif*, dehuman*, OR marginaliz* | AND |
| women, woman, female, OR gender. |  |
