# Supplementary material for: Intersectional discrimination and its impact on Asian American women's mental health: A mixed-methods scoping review
Source: Front Public Health. 2023 Feb 27;11:993396. doi: 10.3389/fpubh.2023.993396 (PMC10008964; doi:10.3389/fpubh.2023.993396)
Supplement: Supplementary file 2 [file Table_2.docx]

**Supplemental Table 2 Characteristics of study sample (N=23)**

| **Author(s) and year** | **Study population^a^** | **Intersectional framework** | **Type of discrimination assessed or identified^b^** | **Level of discrimination^c^** | **Study method** | **Sample Notes** |
| --- | --- | --- | --- | --- | --- | --- |
| Ahn et al., 2021 | Second-generation Asian American women | Intersectionality as explicit framework | Gendered racial discrimination | Unspecified but results suggest multiple levels | Qualitative | Participants were between 20-29 years old |
| Bécares & Zhang, 2018 | Black, Chinese American, Japanese American, and white older women | Intersectionality described in introduction but not explicitly stated to be study framework | Accumulation of discrimination across attributes, domains, and time | Interpersonal | Quantitative | Participants were between 42-52 years old |
| Bhatt, 2013 | First- and second-generation Indian female and male physicians | Intersectionality described in introduction but not explicitly stated to be study framework | Separately assessed gender discrimination and gendered racial discrimination | Unspecified but results suggest multiple levels | Qualitative |  |
| Brady et al., 2017 | Asian American undergraduate women | Intersectionality as explicit framework | Intersection of racism and sexism | Unspecified but results suggest multiple levels | Qualitative |  |
| Buchanan et al., 2018 | Asian American women | Intersectionality as explicit framework | Racial and sexual harassment | Interpersonal | Quantitative | Participants were all undergraduates at a Midwestern university |

**Supplemental Table 2 Continued**

| **Author(s) and year** | **Study population^a^** | **Intersectional framework^b^** | **Type of discrimination assessed or identified^c^** | **Level of discrimination^d^** | **Study method** | **Sample Notes** |
| --- | --- | --- | --- | --- | --- | --- |
| Castillo et al., 2020 | Black-Asian American women and men | Intersectionality described in introduction but not explicitly stated to be study framework | Internalized racism | Authors specified internalized but results suggest multiple levels | Qualitative |  |
| Castro & Collins, 2021 | Asian American women in STEM | Intersectionality as explicit framework | Discrimination at the intersection of gender, race, and science | Structural, institutional, interpersonal | Qualitative | Participants were either doctoral students or had graduated with their PhDs within the past 5 years |
| Endo, 2015 | Asian American female public-school teachers in the Midwest | “Intersections” discussed throughout without referring explicitly to intersectionality framework | Racial microaggressions intersecting with gender dynamics | Authors specified microaggressions (interpersonal) but results suggest multiple levels | Qualitative |  |
| Gamst et al., 2021 | Asian American women | Intersectionality as explicit framework | Racial discrimination, gendered racial microaggressions | Interpersonal | Quantitative |  |
| Gamst et al., 2022 | Asian American women | Intersectionality as explicit framework | Racism-related stress | Interpersonal | Quantitative |  |

**Supplemental Table 2 Continued**

| **Author(s) and year** | **Study population^a^** | **Intersectional framework^b^** | **Type of discrimination assessed or identified^c^** | **Level of discrimination^d^** | **Study method** | **Sample Notes** |
| --- | --- | --- | --- | --- | --- | --- |
| Iwasaki et al., 2016 | Japanese American women married to white men | Not mentioned | Societal microaggressions | Authors specified microaggressions (interpersonal) but results suggest multiple levels | Qualitative | Participants were 47-70 years old |
| Keum et al., 2018 | Emerging adult Asian American women | Intersectionality as explicit framework | Gendered racial microaggressions, racial microaggressions, sexism, internalized racism | Interpersonal, internalized | Quantitative |  |
| Keum et al., 2022 | Asian American women | Intersectionality mentioned in discussion | Gendered racial microaggressions, internalized racism | Interpersonal, internalized | Quantitative |  |
| Kim et al., 2011 | Asian/Asian American women faculty of evangelical Christian belief at a Christian University | Intersectionality described in introduction but not explicitly stated to be study framework | Discrimination in academia | Unspecified but results suggest multiple levels | Qualitative |  |

**Supplemental Table 2 Continued**

| **Author(s) and year** | **Study population^a^** | **Intersectional framework^b^** | **Type of discrimination assessed or identified^c^** | **Level of discrimination^d^** | **Study method** | **Sample Notes** |
| --- | --- | --- | --- | --- | --- | --- |
| Le et al., 2020 | Asian American women | Intersectionality as explicit framework | Gendered racial microaggressions, racism, sexism | Interpersonal | Quantitative |  |
| Lerner & Lee, 2021 | Trans and gender diverse Asian Americans | Intersectionality mentioned in discussion | Discrimination and violence | Institutional, interpersonal | Quantitative |  |
| Liang & Peters-Hawkins, 2017 | Asian American women working in public school administration | Intersectionality as explicit framework | Gender, racial/ethnic, and cultural discrimination in the workplace | Unspecified but results suggest multiple levels | Qualitative |  |
| Mukkamala & Suyemoto, 2018 | Asian American women | Intersectionality described in detail and study aim was to identify “intersectional discrimination” but not explicitly stated to be study framework | Intersectional discrimination | Unspecified but results suggest multiple levels | Qualitative |  |
| Noh, 2018 | Asian American women | Intersectionality briefly mentioned in discussion | Model minority myth | Author specified internalized and structural but results also suggest institutional and interpersonal | Qualitative |  |

**Supplemental Table 2 Continued**

*Notes*. a) Some study samples were limited to a particular age range, despite study aims. See Sample Notes column for caveats on study population. b) Studies that used intersectionality as the framework had to explicitly state that it was the conceptual theory or framework for their study and not just describe it as relevant. c) Type of discrimination assessed or identified uses the language from the study. d) Many of the studies did not specify the level of discrimination assessed or identified. For those that did not, we identified what levels were assessed based on the quantitative measures or the qualitative findings.

| **Author(s) and year** | **Study population^a^** | **Intersectional framework^b^** | **Type of discrimination assessed or identified^c^** | **Level of discrimination^d^** | **Study method** | **Sample Notes** |
| --- | --- | --- | --- | --- | --- | --- |
| Subedi & Maleku, 2021 | Bhutanese-Nepali young women | Intersectionality described in introduction but not explicitly stated to be study framework | Racism | Unspecified but results suggest interpersonal | Qualitative |  |
| Sue et al., 2007 | Asian Americans | Not mentioned | Racial microaggressions | Authors specified microaggressions (interpersonal) but results suggest multiple levels | Qualitative |  |
| Sung et al., 2015 | Lesbian and bisexual Asian American women | Intersectionality described in introduction but not explicitly stated to be study framework | Racism, sexism, heterosexism | Unspecified but results suggest multiple levels | Qualitative |  |
| Wong et al., 2017 | Asian American women | Not mentioned | Stereotypes | Authors specified interpersonal but results suggest multiple levels | Qualitative | Participants were 20-28 years old |
